# Supplementary material for: Determination of the Protein-Protein Interactions within Acyl Carrier Protein (MmcB)-Dependent Modifications in the Biosynthesis of Mitomycin
Source: Molecules. 2021 Nov 10;26(22):6791. doi: 10.3390/molecules26226791 (PMC8621148; doi:10.3390/molecules26226791)
Supplement: Supplementary file 1 [file molecules-26-06791-s001.zip › molecules-1380848-supplementary.pdf]

molecules

Determination of the Protein-Protein Interactions Within Acyl Carrier Protein (MmcB) Dependent Modifications in the Biosynthesis of Mitomycin

Dongjin Leng [1], Yong Sheng[1], Hengyu Wang[1], Jianhua Wei[1], Yixin Ou[1], Zixin Deng[1], Linquan Bai[1] and Qianjin Kang[1,*]

1 State Key Laboratory of Microbial Metabolism, Joint International Research Laboratory of Metabolic & Developmental Sciences, and School of Life Sciences & Biotechnology, Shanghai Jiao Tong University, Shanghai 200240, China; dongjinleng@163.com (D.L.); John.Sheng@sjtu.edu.cn (Y.S.); wanghengyu0203@163.com (H.W.); weijianhua@sjtu.edu.cn (J.W.); yixinou@sjtu.edu.cn (Y.O.); zxdeng@sjtu.edu.cn (Z.D.); bailq@sjtu.edu.cn (L.B.)

* Correspondence: qjkang@sjtu.edu.cn (Q.K); Tel./Fax: +86-21-3420-6119

Supplementary Tables

Table S1. Strains and plasmids used in this study

Strains and plasmids	Description	Source or reference
Streptomyces sp.		
Strain NRRL 2564	Wild-type producer for mitomycin C	[1]
DJ01	Gene *mitE*-inactivation mutant	This work
DJ01::*mitE*	DJ01 complemented with cloned *mitE*	This work
DJ03	Gene *mmcB*-inactivation mutant	This work
DJ03::*mmcB*	DJ03 complemented with cloned *mmcB*	This work
DJ03::*mmcB* (S43A)	DJ03 complemented with cloned *mmcB* (S43A)	This work
DJ05	Gene *mitB*-inactivation mutant	This work
DJ05::*mitB* (originated)	DJ05 complemented with cloned *mitB* (originated)	This work
DJ07::*mitB* (updated)	DJ05 complemented with cloned *mitB* (updated)	This work
DJ08	Gene *mitF*-inactivation mutant	This work
DJ08::*mitF*	DJ08 complemented with cloned *mitF*	This work
Escherichia coli		
E. coli DH10B	Cloning host	Invitrogen
E. coli ET12567/pUZ8002	Conjugation donor strain	[2]
E. coli BL21 (DE3)	Protein expression strain	Invitrogen

E. coli BL21(DE3)/pSV20	Protein expression strain containing of *sfp*	[3]
Plasmids		
pBluescript SK (+)	*ColE*, *lacZ*, *bla*, *orif1*	Stratagene
pJTU1278	*rep pIJ101, tsr, bla, oriT*	[4]
pSET152	*ΦC31, oriT, kasO*p, acc(3)IV*	[5]
pET28a	*kan*, P_{T7}, His_6-tag	Novagen
pJQK401	Construct for *mitE* deletion	This work
pJQK402	pSET152 with cloned *mitE*	This work
pJQK406	pET28a with cloned *mitE*	This work
pJQK408	pSET152 with cloned *mitB* (originated)	This work
pJQK409	pSET152 with cloned *mitB* (updated)	This work
pJQK411	pET28a with cloned *mitB* (updated)	This work
pJQK412	Construct for *mitF* deletion	This work
pJQK413	pSET152 with cloned *mitF*	This work
pJQK414	pET28a with cloned *mitF*	This work
pJQK415	pET28a-TEV with cloned *mmcB*	This work
pJQK416	pET28a-TEV with cloned *mitB*	This work

Table S2. Primers used in this study

Primers	Sequences (5′→3′)
mitE-petF	ATACATATGACTGAACAGGCGACCGGTC, *Nde*I site underlined
mitE-petR	ATAGAATTCATGAACCGGCCTCCTTGGC, *Eco*RI site underlined
mitB-petF	ATA CATATGCGGGCCCCCAACGGCACC, *Nde*I site underlined
mitB-petR	ATAGAATTCATCGCGCCGCGTCCCGTGC, *Eco*RI site underlined
mmcB-petF	ATACATATGGAGACCCTGACGACCGA, *Nde*I site underlined
mmcB-petR	ATAGCGGCCGCTCATTCGGCGGGTGCGGT, *Not*I site underlined
mitB-left armF	ACGGGATCCCAGGACCGAAAGGCTGCTCAATG, *Bam*HI site underlined
mitB-left armR	ATGAAGCTTCAGGACGTCGTCGTCGAGGAAGA, *Hin*dIII site underlined
mitE-left armF	AAAGGTACCCCACTCATGTCCCGTAGCACCC, *Kpn*I site underlined
mitE-left armR	AAGAAGCTTGGTCAACGTCAGGCGGAGGC, *Hin*dIII site underlined
mmcB-left armF	ACGGGATCCGGCGGTCACGGGATCTATCA, *Bam*HI site underlined
mmcB-left armR	GGGAAGCTTGAGGGAATCGACGAGCACCTT, *Hin*dIII site underlined
mitB-right armF	CCCAAGCTTCACCACCGCAACCTCACGCACT, *Hin*dIII site underlined

mitB-right armR	CCGGTACCCGACCTTCTCGTGCAGGCTCCC, *Kpn*I site underlined
mitE-right armF	CCCAAGCTTCTGGTCGACGACGTCTTCCTGC, *Hin*dIII site underlined
mitE-right armR	GCGGATCCAGCCCGACACTGCGCACGGT, *Bam*HI site underlined
mmcB-right armF	CCCAAGCTTATCGACAGTCTCGACCTCCTCGC, *Hin*dIII site underlined
mmcB-pdF	CAGCATATGGAAACCCTGACCACCGA, *Nde*I site underlined
mmcB-pdR	CTCGAATTCATTCCGCCGGTGCGGTAACG, *Eco*RI site underlined
mitB-pdF	CAGCATATGCGGGCCCCCAACGGCA, *Nde*I site underlined
mitB-pdR	CTCGAATTCATCGCGCCGCGTCCCGTGCCGGT, *Eco*RI site underlined
mitF -petF	CCCATATGAGCACCGTCACCGACCGG, *Nde*I site underlined
mitF -petR	CG GAATTC TCAGAAGCGGGCACCACCG, *EcoRI* site underlined

Supplementary Figures

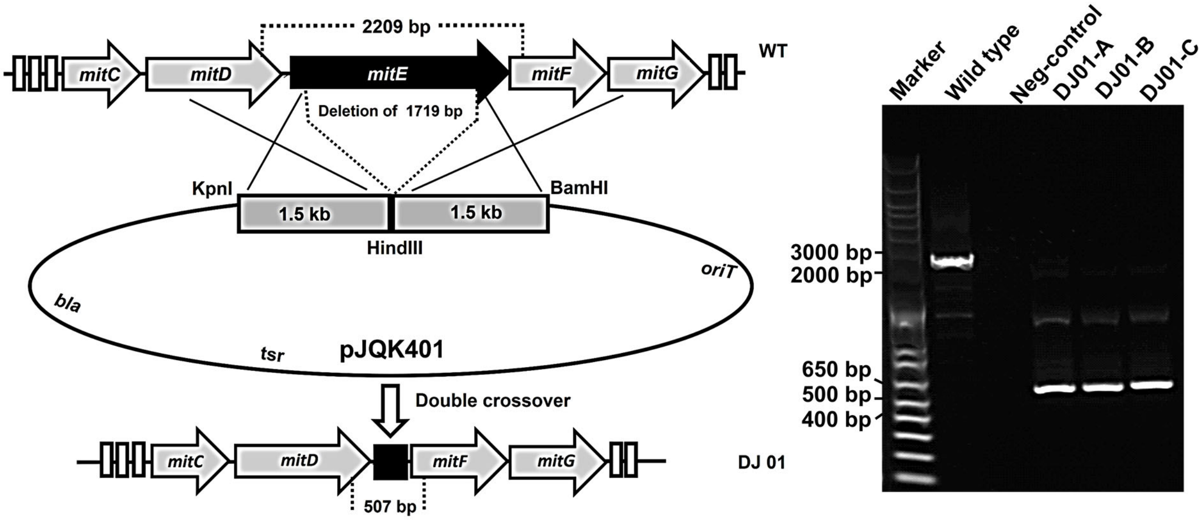

Figure S1. The construction of DJ01 mutant. (A). Schematic representation of the in-frame deletion of *mitE*, a 1,719-bp region of *mitE* was deleted through double crossover. (B). Confirmation of the mutant DJ01 through PCR amplification. An approximately 500-bp product was amplified for the DJ01, while a 2,200-bp fragment was obtained from the wild type with the no template-added reaction as the negative control.

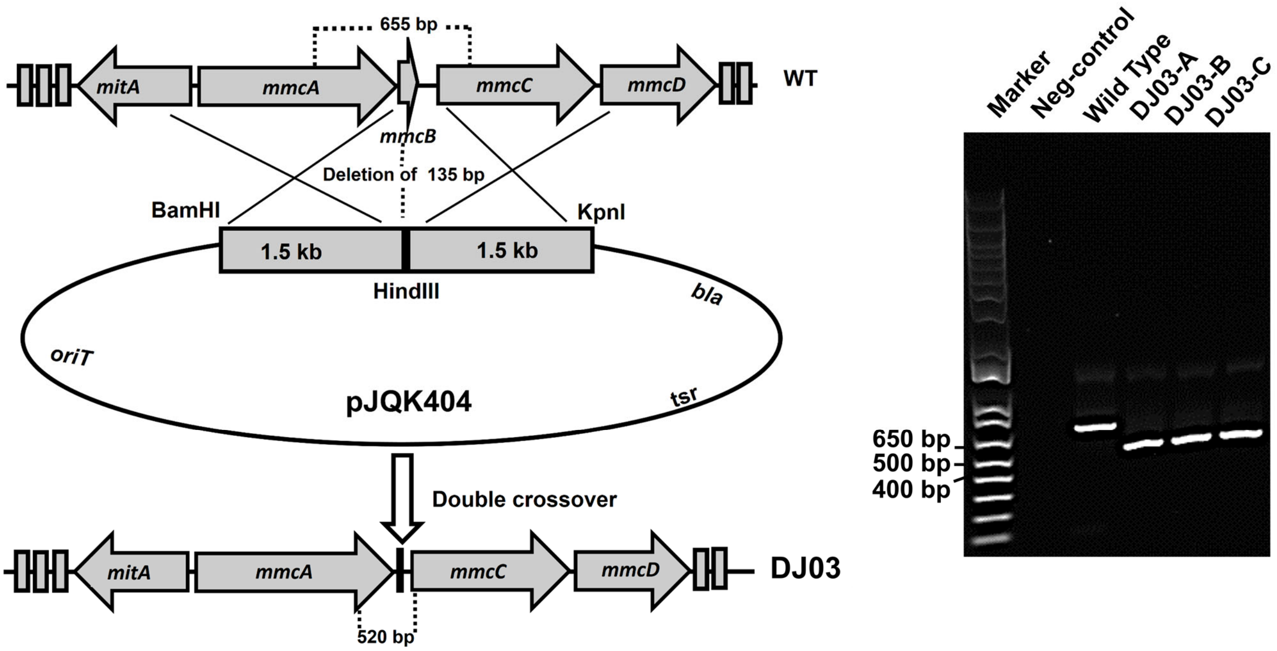

Figure S2. The construction of DJ03 mutant. (A). Schematic representation of the in-frame deletion of *mmcB*, a 135-bp region of *mmcB* was deleted through double crossover. (B). Confirmation of the mutant DJ03 through PCR amplification. An approximately 520-bp product was amplified for DJ03, while a 650-bp product was obtained from the wild type with the no template-added reaction as the negative control

A.

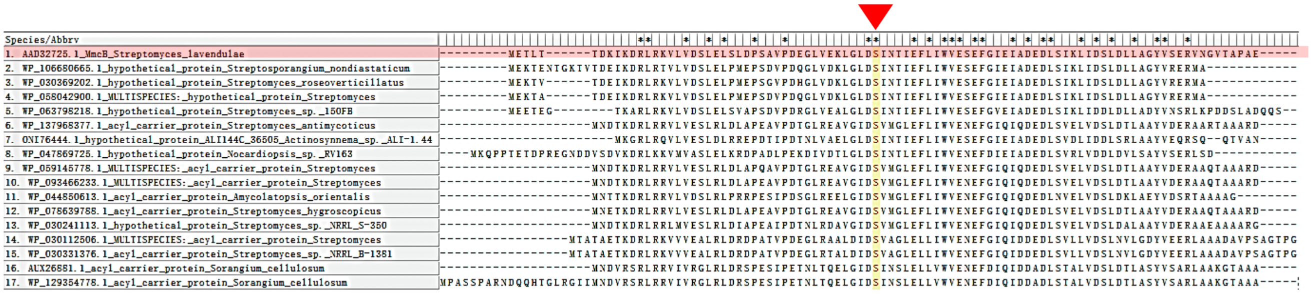

B.

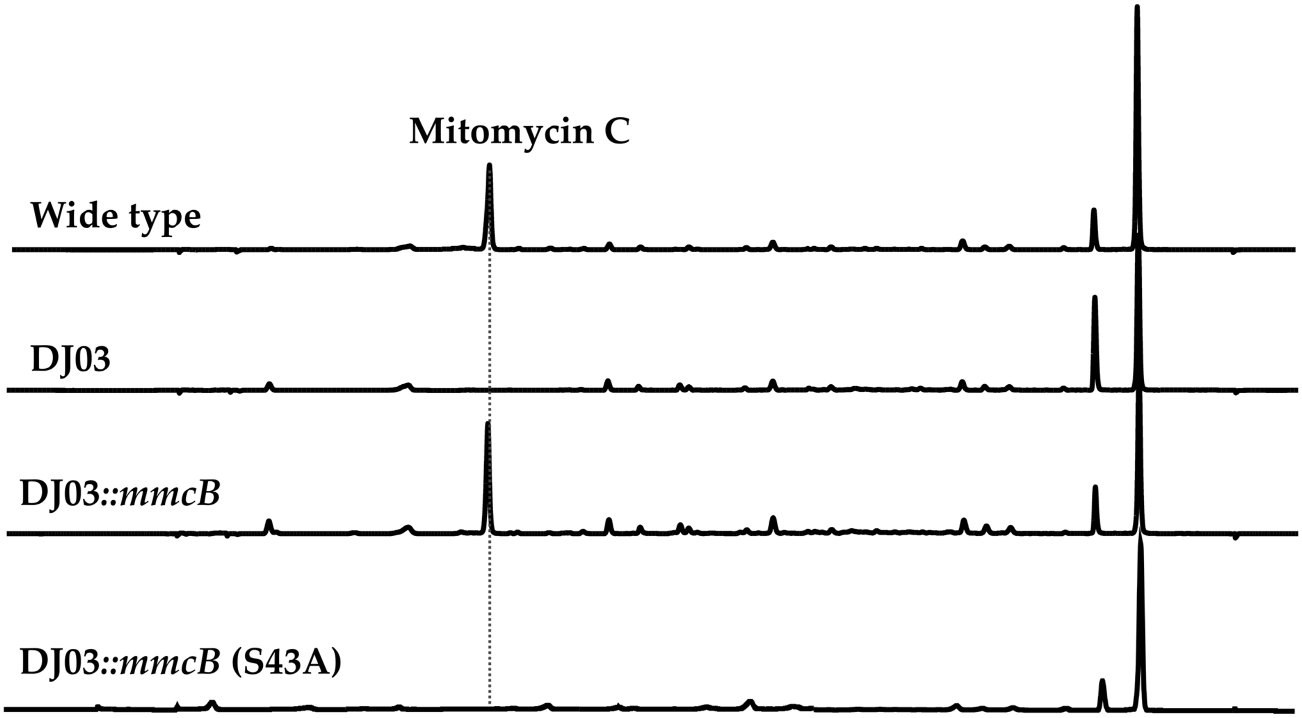

Figure S3. Determination of the active site of MmcB. (A) Sequence alignment of MmcB with the homologies, (B) HPLC analysis results of *mmcB* and *mmcB* S43A complementation experiments revealed the active site of Ser 43 residue.

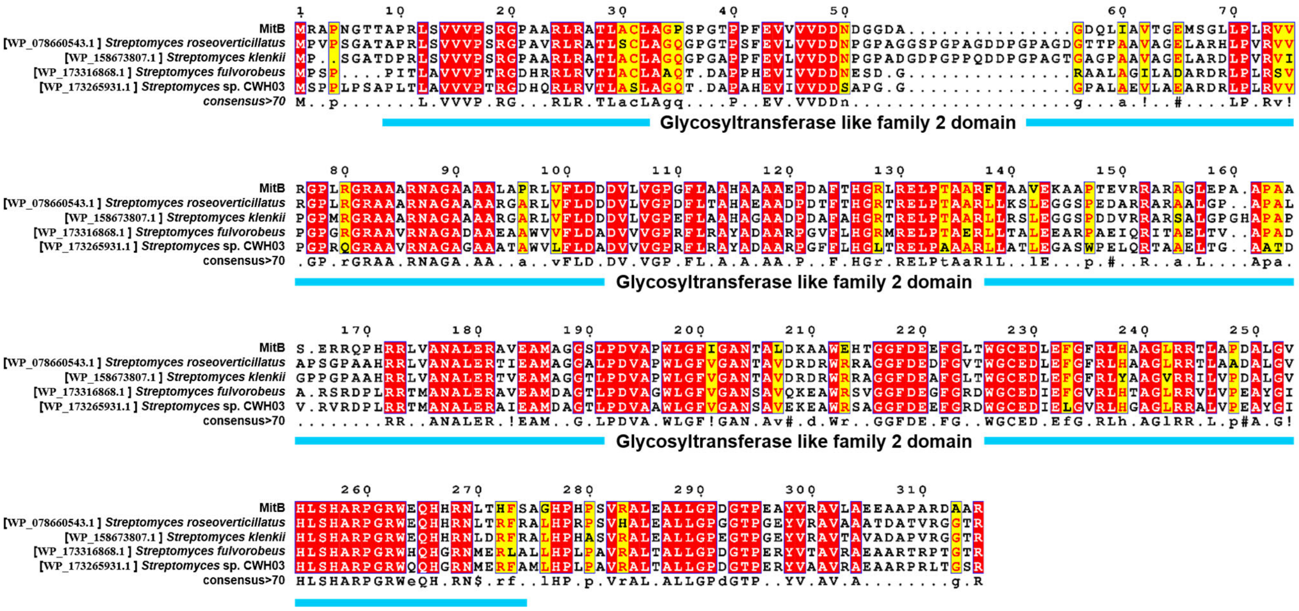

Figure S4. The sequence alignment of MitB with its homologies. The targeted family 2 glycosyltransferases including of WP_078660543.1 from *Streptomyces roseoverticillatus*, WP_158673807.1 from *Streptomyces klenkii*, WP_173316868.1 from *Streptomyces fulvorobeus*, and WP_173265931.1 from *Streptomyces* sp. CWH03.

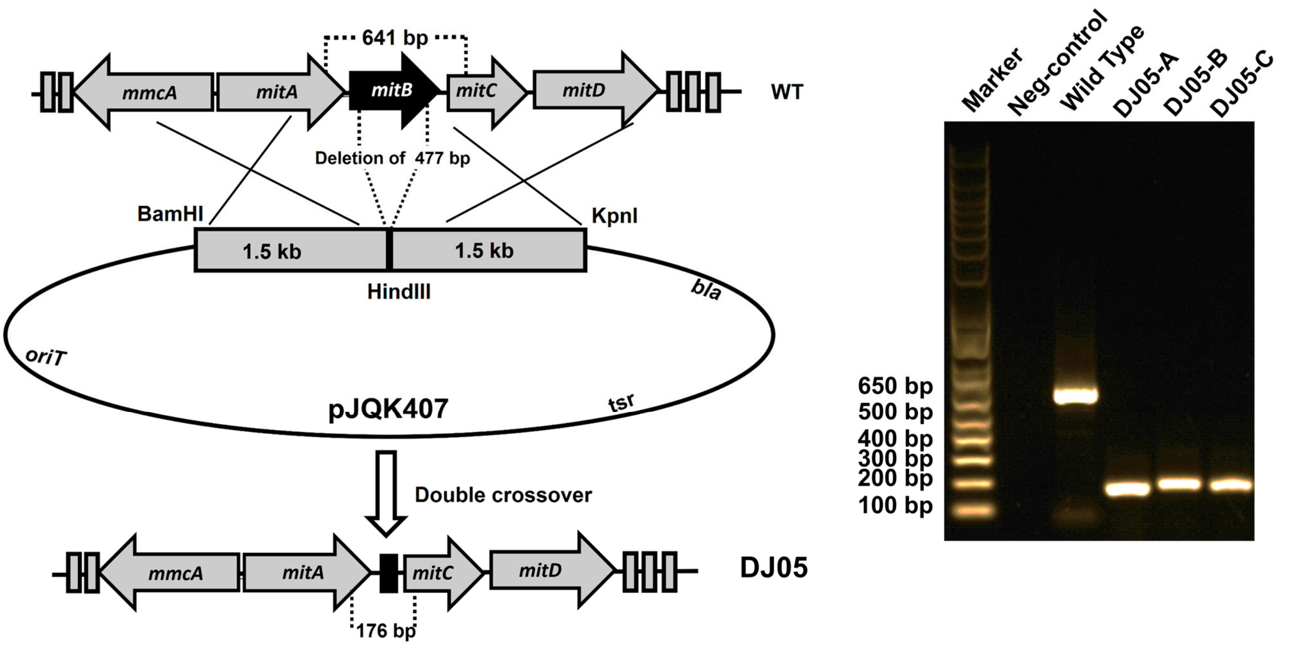

Figure S5. The construction of DJ05 mutant. (A). Schematic representation of the in-frame deletion of *mitB*, a 477-bp region of *mitB* was deleted through double crossover. (B). Confirmation of the mutant DJ05 through PCR amplification. An approximately 170-bp product was amplified for DJ03, while a 640-bp product was obtained from the wild type with the no template-added reaction as the negative control.

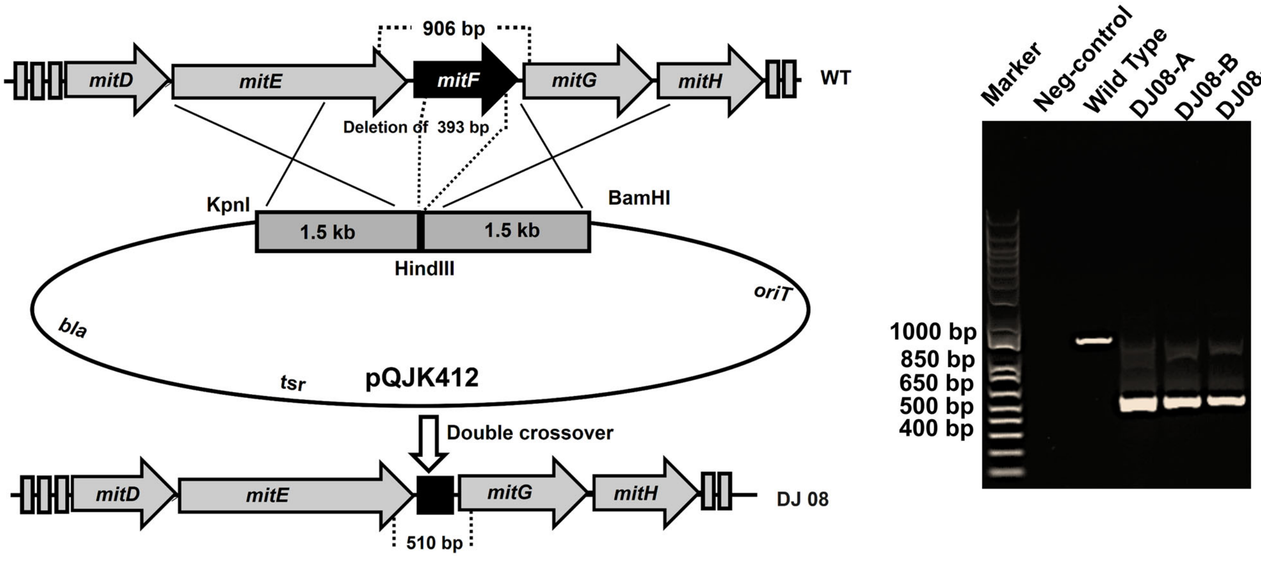

Figure S6. The construction of DJ08 mutant. (A). Schematic representation of the in-frame deletion of *mitF*, a 393-bp region of *mitF* was deleted through double crossover. (B). Confirmation of the mutant DJ08 through PCR amplification. An approximately 510-bp product was amplified for DJ03, while a 906-bp product was obtained from the wild type with the no template-added reaction as the negative control.

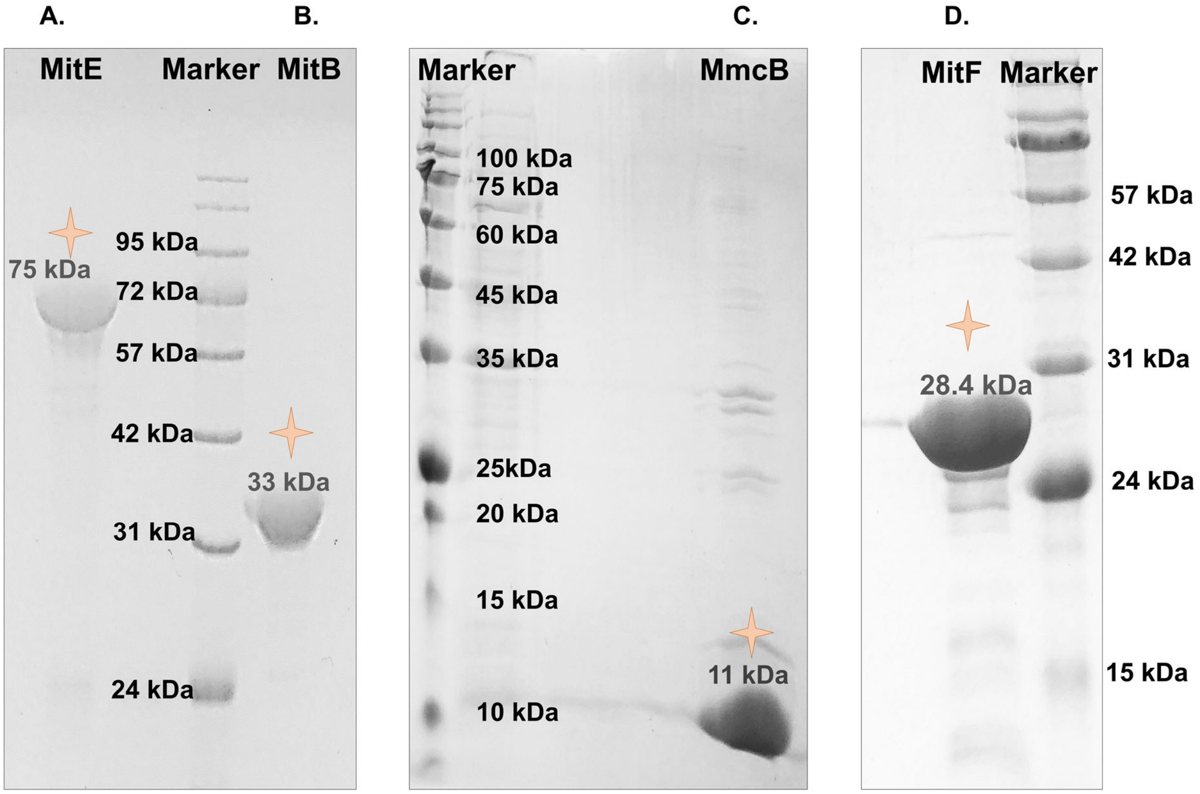

Figure S7. SDS-PAGE analysis of the purified MitE (A), MitB (B), MmcB (C) and MitF (D) expressed in *E. coli* BL21(DE3)

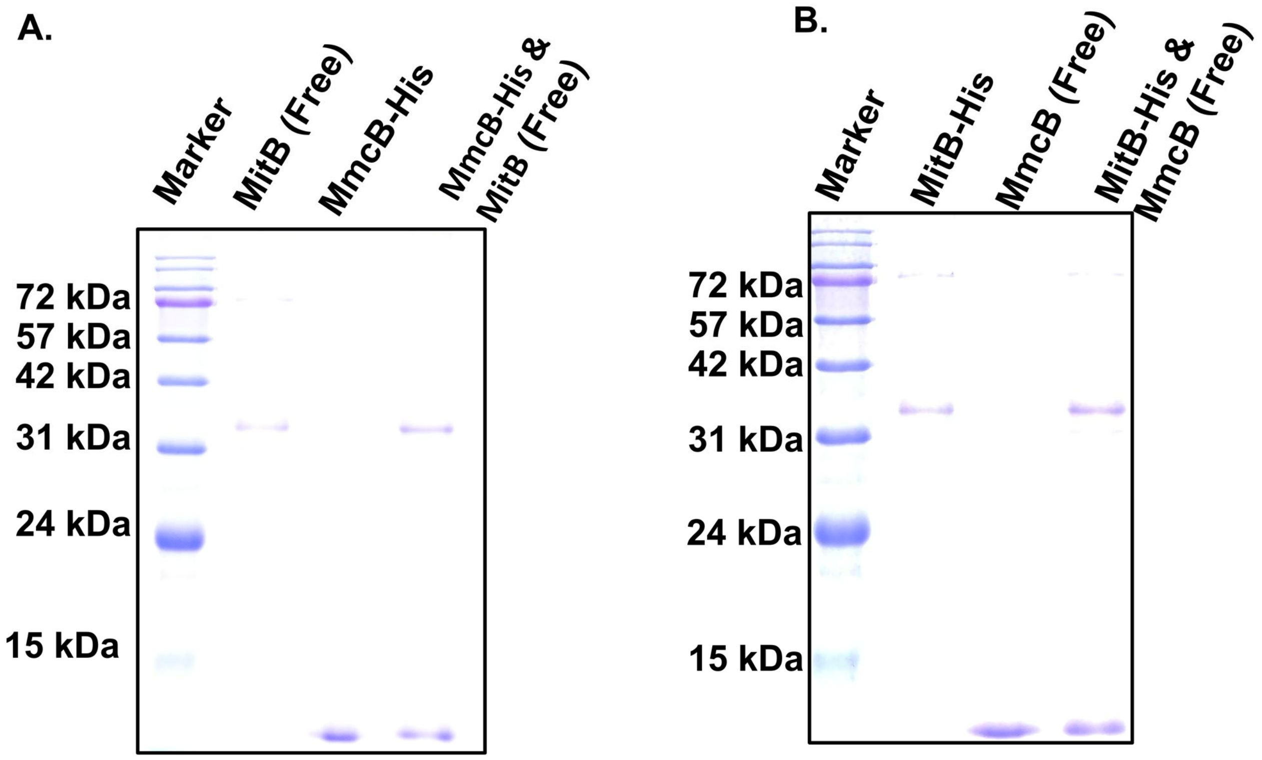

Figure S8. The *in vivo* pull-down assay between MmcB and MitB

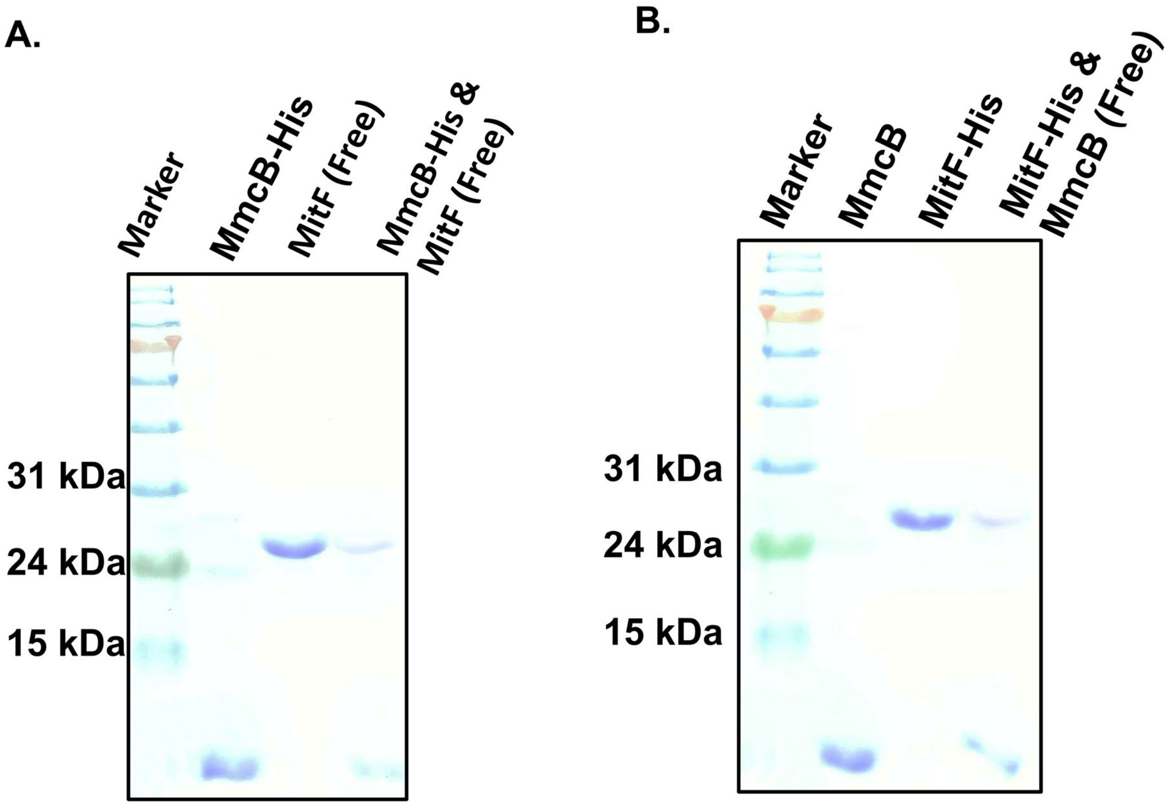

Figure S9. The *in vivo* pull-down assay between MmcB and MitF

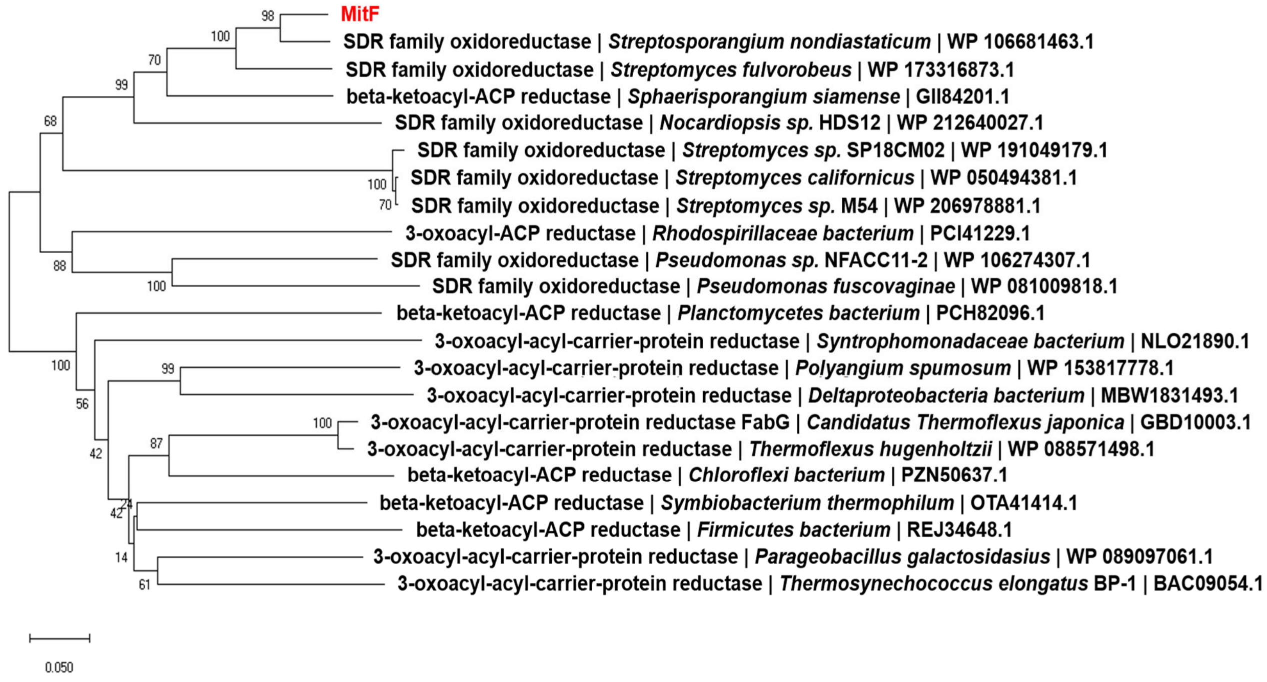

Figure S10. Phylogenetic analysis of MitF with its homologous. The analysis was performed in MEGA 11 software. The amino acid sequences alignment of MitF and homologs were conducted by using ClustalW, and then phylogenetic analyses were further proceeding by the neighbor-joining method. The parameters of phylogeny were set as follows: bootstrap test (1000 replicates), *p*-distance mode, and complete deletion. The numbers adjacent to branches represent bootstrap values.

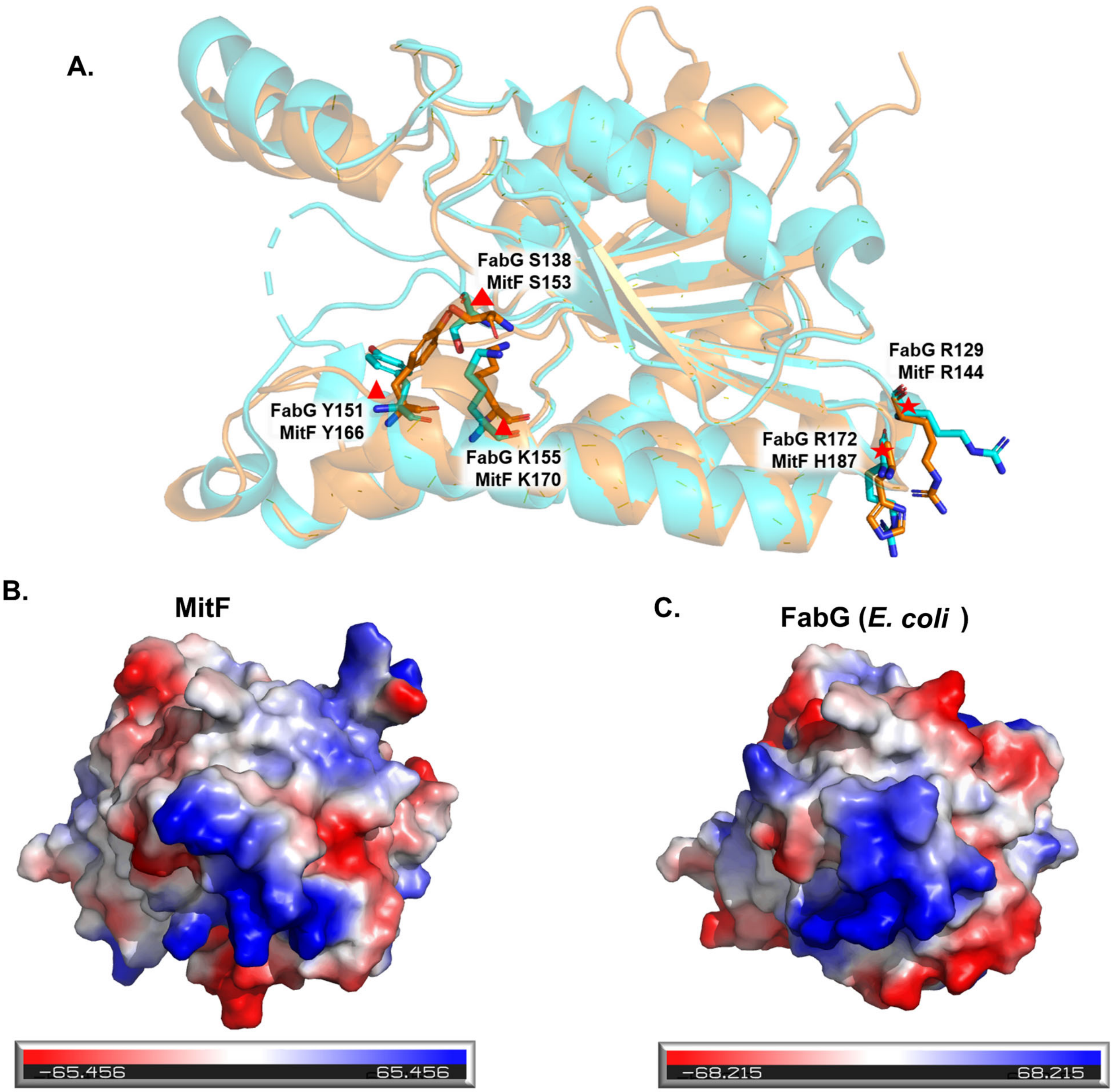

Figure S11. The models of MitF with the FabG (PDB: 1Q7B) by SWISS-MODEL server. (**A**) The superimposition of the MitF with FabG revealed the conserved activity center, (**B**) Characteristic electron cloud map of the MitF, (**C**) Characteristic electron cloud map of the FabG.

References

1. Mao, Y.; Varoglu, M.; and Sherman, D.H. Molecular characterization and analysis of the biosynthetic gene cluster for the antitumor antibiotic mitomycin C from *Streptomyces lavendulae* NRRL 2564. *Chem. Biol.*, **1999,** *6*, 251-63.
2. Paget, M.S.; Chamberlin, L.; Atrih, A.; Foster, S. J.; Buttner, M. J. Evidence that the extracytoplasmic function sigma factor *sigmaE* is required for normal cell wall structure in *Streptomyces coelicolor* A3(2). *J. Bacteriol.*, **1999,** *181*, 204-11.
3. Zhang, D.; Zhang, F.; and Liu, W. A KAS-III heterodimer in lipstatin biosynthesis nondecarboxylatively condenses C(8) and C(14) fatty acyl-coa substrates by a variable mechanism during the establishment of a C(22) aliphatic skeleton. *J. Am. Chem. Soc.*, **2019**, *141*, 3993-4001.
4. He, Y.; Wang, Z.; Bai, L.; Liang, J.; Zhou, X.; Deng, Z. Two pHZ1358-derivative vectors for efficient gene knockout in streptomyces. *J. Microbiol. Biotechnol.* **2010**, 20, 678-82.
5. Bierman, M.; Logan, R.; O'Brien, K.; Seno, E. T.; Rao, R. N.; Schoner, B. E. Plasmid cloning vectors for the conjugal transfer of DNA from *Escherichia coli* to *Streptomyces* spp. *Gene.* **1992**, *116*, 43-49.
